# The Effect of Prior Viewing Position and Spatial Scale on the Viewing of Paintings

**DOI:** 10.3390/vision7030055

**Published:** 2023-08-22

**Authors:** Tobiasz Trawiński, Natalie Mestry, Nick Donnelly

**Affiliations:** 1 Department of Psychology, Liverpool Hope University, Liverpool L16 9JD, UK; donneln@hope.ac.uk; 2 Department of Psychology, Bournemouth University, Poole BH12 5BB, UK; nmestry@bournemouth.ac.uk

**Keywords:** eye movements, memory for paintings, Navon stimuli, starting position, spatial scale

## Abstract

The visual inspection of scenes is disrupted when participants are forced to begin inspection away from the centre of an image. The present study explored the effect of the starting point on the visual inspection of paintings. Eye movements were recorded while participants viewed paintings for later identification in a yes/no discrimination task. The viewing of each painting was preceded by the presentation of a pseudo-randomly positioned Navon figure. Participants were instructed using a cue to attend to either the local or global level of the Navon figure. Each painting was split into regions of interest (ROIs) defined by face, theme, and context to allow the analysis of eye movements. These data were directly compared with a subset of those initially reported in our previous study in which the same experiment was run but without the inclusion of the Navon figure. The inclusion of the Navon task lowered the discrimination accuracy in the yes/no discrimination task. More importantly, eye movements to the paintings were disrupted across the entire period over which they were viewed and not just in the period following the offset of the Navon figure. The results show the sensitivity of eye movements to the conditions present at the beginning of viewing. The results have implications for the viewing of paintings (and other images) in the real world, where the starting conditions for inspection cannot be controlled.

## 1. The Effect of Prior Viewing Position and Spatial Scale on the Viewing of Paintings

Studies of the eye movements made by beholders looking at representational paintings suggest that their fixations are usually distributed around the central area of the painting, especially for those beholders without art training [1,2,3,4,5,6,7,8,9,10,11,12,13]. The tendency for beholders to look at the centre of paintings is reinforced by the artist’s use of colour, luminance, texture, perspective, and the visual weighting of the picture elements [14,15,16,17,18,19,20,21,22,23,24], which come together to strengthen the importance of the central area [25,26].

The compositional aspects of paintings that reinforce the importance of the central area of the painting as a focus for visual inspection may build on a general central fixation bias that seems to influence the inspection of images of natural scenes. So-called central fixation bias is consistently reported across different experimental paradigms [24,27,28,29,30,31,32,33]. Moreover, studies have shown that central fixation bias is unaffected by the distribution of image features across scenes (e.g., [32]). It is especially robust at the beginning of the viewing even when the scenes being inspected illustrate social interactions [34,35].

Though paintings are different from natural scenes, the central fixation bias found with natural scenes is of particular interest in the context of art perception studies. In lab-based studies of the viewing of paintings, participants are usually required to start their viewing from the centre of a painting. This is achieved by introducing the centred fixation point prior to stimulus presentation [2,6,23,36,37,38,39] or by asking participants to start their viewing by positioning themselves in front of the centre of the painting reproduction (e.g., [40,41]). In the real world of the spectatorship of paintings viewed in a gallery setting, beholders cannot ensure that they are positioned in front of the centre of a painting. The presence of others may mean that they must view a painting to the left or right of what might be thought of as an optimal viewing position. While such a viewing position does not preclude fixations to the centre of a painting, it both reduces their probability and influences perspective when they are made. In doing so, it raises the question as to the extent to which the spectatorship of paintings is impacted by beginning viewing away from the centre of a painting.

The following question is considered in the present study: what is the effect on the visual inspection of paintings of starting viewing at a location away from the centre? Central fixation bias is known to be particularly marked when scenes show social interactions. Given that representational paintings often show people and early fixations are often made to people (e.g., [6,23,39,42,43]), it might be that cues that lead to viewing beginning at locations away from the centre may be quickly discounted. However, there is evidence that the influence of manipulating the starting location for viewing may have a long-lasting effect on eye movements. A recent study by Rothkegel et al. [44] investigated to what extent the manipulation of the position of the initial fixation affected subsequent eye movements to images of natural scenes. The surprising result reported by Rothkegel et al. was that starting position influenced viewing behaviour for the first five seconds such that eye movements oscillated across scenes rather than settling in the central area. In the present study, we explored the impact of the starting location for viewing in representational paintings to test its impact on the central fixation bias.

A common attribute of eye movement studies in scene perception, including those that explore the influence of starting position on viewing, is that trials begin with the presentation of a fixation cross. The majority of studies do not consider the fixation cross as impacting viewing beyond establishing an initial time and location for viewing. However, this may be a naïve position to hold in the context of studies on the eye movements made in the viewing of paintings. With respect to scene perception studies, Bindemann [34] showed that central fixation bias could be an artefact that arises in visual perception studies in laboratory settings (see also [33,45]) and it cannot be simply removed by offsetting the scene from the screen centre or varying location of the of proceeding fixation cross [32,35]. Currently, we do not know whether, in the context of studies on the eye movements made in the viewing of paintings, the central fixation bias results from the influence of the fixation cross or the importance of the objects in the central area of the painting. Answering this question would allow us to generalise the importance of central fixation bias beyond scene perception studies.

The fixation cross also has the potential consequence of focusing attention on a local spatial scale [46] given that spectatorship can focus on very local aspects of the painting through the gist of the whole canvas. Typically, when viewing paintings in an art gallery or museum setting, the spatial scale of the initial allocation of attention to a painting is likely to be influenced by that used a prior painting viewing. Consequently, we were concerned that any study of the starting point for viewing should also consider this issue and manipulate the spatial scale adopted by the viewer at the beginning of inspection. In the present study, both the spatial scale at the beginning of viewing and the starting location for viewing were varied such that participants were required to memorise for later report the global or local identity of a pseudo-randomly positioned Navon stimulus ([47,48] see [49] for review) presented prior to the presentation of paintings. Attending to the global feature of the Navon stimulus can influence of holistic processing of faces [50,51,52] and words (e.g., [53,54]). Perhaps more pertinently, responding to a global Navon stimulus prior to the presentation of a scene is known to influence the features attended when categorising scenes (e.g., [55]). Specifically, attending the global Navon feature increased the likelihood of using low spatial frequency content. In the present study, we hypothesised that inspecting the paintings after responding to the global feature of the Navon stimulus may increase the likelihood of using information away from the centre.

To test the hypothesis that the spatial scale and starting point for viewing influence eye movements made in the inspection of paintings, it is possible to just compare the pattern of distribution of fixations across paintings and conditions. However, our previous research showed the importance of considering the eye movements made to paintings with respect to three regions of interest (ROIs): faces, the area detailing a painting’s primary theme and its context [39,56]. The theme is usually in the geometric centre of the painting, placed in the foreground, and presents the core narrative of the painting (which typically focuses on human figures). The context surrounds the theme. In practice, while the relationship between theme and context may seem open to interpretation, art experts strongly agree on what areas constitute theme and context [25,57,58]. The theme accords with where one might expect a central fixation bias to be found. Faces are predominantly present in the theme and are known to attract fixations, especially during early viewing [23]. For this reason, they are a particularly critical measure of the manipulation of viewing conditions.

Here, we asked participants to memorise the same set of paintings as used in Trawiński et al. ([39], see also [56] for similar approaches) for the yes/no discrimination task performed after the encoding session. The use of this image set meant that we also had a body of published data reporting the viewing strategy when participants were given a centrally positioned fixation cross at the start of viewing. In the present study, we began by exploring the impact of participants’ spatial scale and the starting location of their viewing on the distribution of fixations. We predicted that the spatial scale and a starting point of viewing away from the centre of the painting would impact central fixation bias. In disrupting the central fixation bias, we expected that participants would tend to make more fixations on the context and theme rather than the face ROI. This hypothesis was tested by comparing the spatial distribution of eye movements made to ROIs during the encoding of the new participants with those presented in Trawiński et al. [39]. Next, we compared the spatial distribution of fixations across viewing time. We do this to examine whether any effects of the spatial scale and starting location on the spatial distribution of fixations were limited to the immediate onset of the painting or extended for the full duration of viewing.

Finally, we also tested the impact of the inclusion of the Navon figure prior to painting viewing on eye movements. We predicted that the tendency to attend to the context relative to the other ROIs would be increased by the presence of the global Navon figure prior to painting viewing. To examine the magnitude of this effect, we compared the likelihood of fixating on each ROI in local and global conditions across viewing time.

## 2. Method

### 2.1. Participants

Data were gathered from 31 undergraduate students (5 males and 20 females; *M_age_* = 23.80, *SD_age_* = 9.20) from the University of Bournemouth who took part in the study for course credits or payment (GBP 15). Expertise in art was measured with an art knowledge questionnaire. Participants’ knowledge about art was in the lower half of the possible answers (range 2–16 out of 48, see Table 1). The participants were, therefore, classified as naïve. The sample size was based on the previous studies that examined the spatial distribution of eye movements made to paintings [2,5,23].

As the data of the new group of participants were to be compared with those previously reported in Trawiński et al. [39], we sought evidence of the comparability of the new group of participants with the 39 British participants reported in Trawiński et al. [39] in a set of basic perceptual and cognitive tests. A two-sample *t*-test confirmed that the participants tested in the present study did not differ from those reported on in Trawiński et al. [39] in executive attention, alerting, or orienting attention (as measured by the Attention Network Task (ANT), *t*(68) = −0.38, *p* = 0.709, *d* = −0.09; *t*(68) = 0.82, *p* = 0.413, *d* = 0.20; *t*(68) = 1.35, *p* = 0.180, *d* = 0.33, respectively; see Table 1), inhibitory attention (as indexed by saccadic latency in an anti-saccade task, *t*(68) = −0.87, *p* = 0.388, *d* = −0.21; *t*(68) = −0.22, *p* = 0.828, *d* = −0.05; 1 s and 3 s delayed conditions, respectively) and spatial and verbal working memory (as measured by the 3-back task, *t*(63.74) = −1.59, *p* = 0.118, *d =* −0.36; *t*(59.07) = 0.08, *p* = 0.935, *d* = 0.02).

### 2.2. Apparatus

Tasks were presented on an Iiyama Vision Master Pro 510 monitor with a screen size of 40 cm by 30 cm, and eye movements were measured using an SR Research Limited Eye-Link 1000 eye tracker operating at 1000 Hz. Viewing was binocular, though only movements of the right eye were recorded. Head movement was stabilised using a chin- and headrest. Participants were seated 70 cm from the screen, resulting in a visual angle of 31.89° by 24.19°. The screen resolution was 1024 × 768 with a refresh rate of 120 Hz. Participants responded by pressing one button on a five-button response box.

### 2.3. Stimuli

The set of 150 images of Western representational paintings used in Trawiński et al. [39] (see Table S1) were used. The height of the paintings varied between 199 and 697 pixels on the screen, resulting in visual angles between 6.35° and 21.79°. Widths varied between 509 and 910 pixels, that is, the visual angles were from 16.17° to 28.48°. Paintings were always presented centrally on the screen against a grey background.

**Figure 1:**
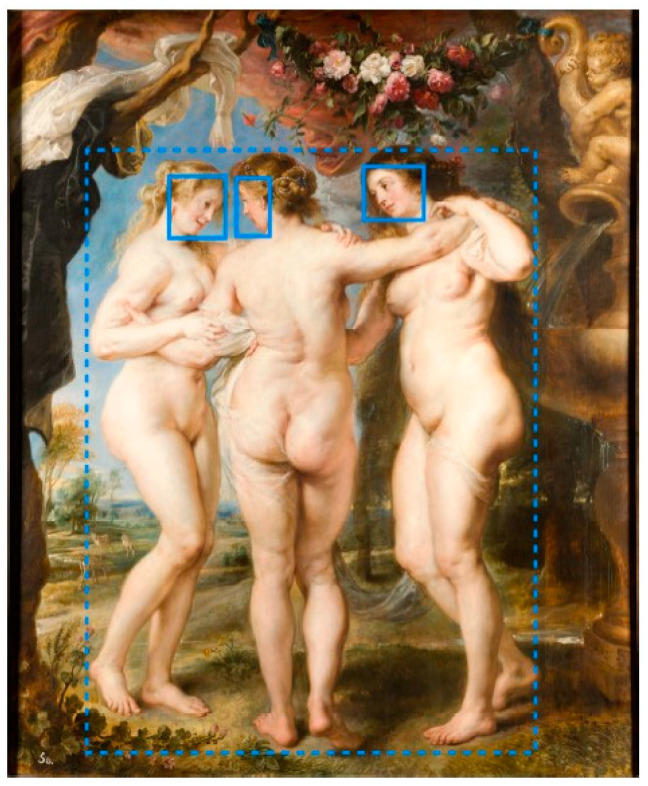
The Three Graces (Peter Paul Rubens, c. 1630). Note. In this example, the motif is Three Graces, so the theme ROI includes three figures (dashed line). The face (solid line) and context (remaining area of the painting) ROIs are also shown.

Face, theme, and context ROIs were defined in each painting. The face, theme, and context ROIs covered, on average, 3%, 55%, and 42% of the area of paintings. For further details on how this was achieved, please see Trawiński et al. [39] and Trawiński, Zang et al. [56]. See Figure 1 for an example of how ROIs were defined for one painting.

**Figure 2:**
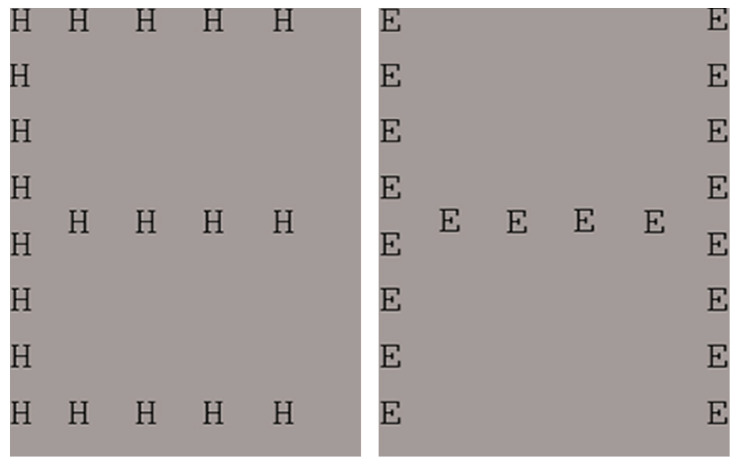
The incongruent Navon stimuli used in the present study.

The Navon stimuli were created in Adobe Photoshop CS6. Twenty letters (E or H) made an incongruent global letter (i.e., an E made out of Hs or an H made out of Es; see Figure 2). The size of the global letter (256 × 341 pixels) was 20 times as large as the smaller local letters (12.8 × 17.05 pixels). The visual angle was 13.52° to 13.51° for width and height, respectively. The letters were presented in black on a grey background. The grey colour matched the background on which the paintings were presented (see Figure 2).

### 2.4. Design and Procedure

The experiment had five stages, as described in Trawiński et al. [39]. The full details of every stage of the present study can be found there. Here, we provide an overview of the key information along with the five-stage procedure that was not included in Trawiński et al. [39].

In the first stage, participants were asked to fill in an art knowledge questionnaire [23]. The art knowledge questionnaire had two parts. In the first part, participants were presented with a list of names of ten artists (e.g., Any Warhol) and asked to indicate whether they recognised the name (yes or no response), state the nationality of the artist (e.g., American), and describe the artistic style in which they worked (ready-made/pop-art). In the second part, participants were presented with six examples of the artwork (e.g., S. Dali’s *Soft Construction with Boiled Beans (Premonition of Civil War)*, 1936) and asked to indicate whether they were familiar with the artwork (yes or no response), state the name of the artist creating it (e.g., Salvador Dali), and describe the style the artwork represented (e.g., surrealism). The maximum score was calculated as the sum of all ‘yes’ and correct responses. The total possible score was 48.

**Figure 3:**
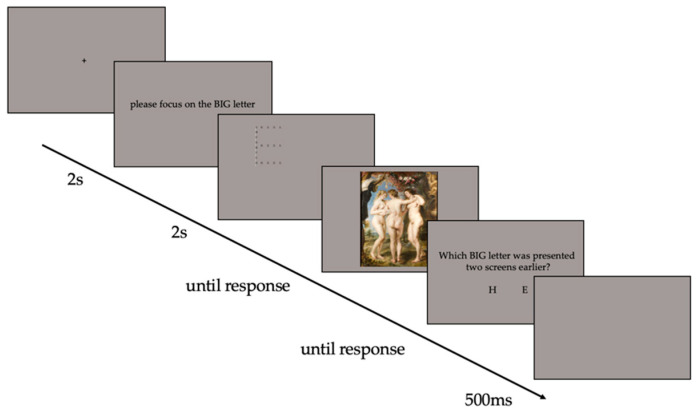
The trial structure at the encoding stage.

In the second stage (the encoding stage), participants attempted to memorise one hundred paintings. The encoding stage was proceeded by a nine-point calibration procedure for accurate eye movement recording. The eye tracker was calibrated to less than 0.5^0^ error. Next, the fixation point was presented in the centre of the screen. Once the fixation point was fixated, one of two possible sentences was presented for 2 s: “*please focus on the BIG letter*” or “*please focus on the SMALL letters*”, representing the global and local conditions, respectively. The location of the Navon stimulus on the screen was pseudo-randomised, i.e., presented in a random location within the screen boundaries, while ensuring that the full figure was always visible on the screen. The pseudo-randomly positioned Navon figure (PRPNF) was presented for 2 s [59] and immediately followed by the presentation of a painting. The cue, with instructions on whether the participant should attend the local or global feature of the Navon figure, was counterbalanced. Participants indicated that they finished viewing by pressing a key on the response box. Next, the participants were asked to report the global or local letter of the PRPNF (either E or H) via two of four buttons on a response box. The blank page was presented for 500 ms during the inter-trial interval (see Figure 3).

In the third stage, participants performed an Anti-Saccade Task [60]. Depending on the identity of the visual cue presented in the centre of the screen, participants either made a saccade to a peripheral stimulus (the prosaccade condition) or generated a saccade to the opposite position (the anti-saccade condition). The inter-stimulus interval between the offset of the cue and the onset of the stimulus was either 1 or 3 s. Varying the inter-stimulus interval introduced uncertainty as to when an eye movement would be required [61].

In the fourth stage (the discrimination stage), participants were shown 100 paintings one at a time and asked to discriminate between the paintings shown in stage 2 (50% of trials) from foils (50% of trials). The same set of paintings was shown to all participants during the discrimination stage. Once the fixation point was fixated, a painting was presented and remained on the screen until a response was made. Responses were made by pressing one of two buttons on a response box. The interval between stages 2 and 4 was around 30 min.

In the fifth stage, participants completed the remaining battery of visual–cognitive measures: the Three-Back Task [62] and the Attention Network Task (ANT; [63]). The Three-Back Task gives a measure of the visual and spatial working memory capacity, and the ANT provides a measure of orienting, alerting, and executive attention.

## 3. Results

We begin by reporting the impact of the encoding conditions on discrimination by comparing the accuracy data from the present (i.e., varied location) experiment with those reported by Trawiński et al. ([39]; i.e., central location). We then compared the accuracy of the performance following the global and local conditions in relation to the new data collected in the varied location study. Together, these analyses explore the overall effect of the starting viewing position and spatial scale on performance in the yes/no discrimination task. The analyses provide an important background to test our hypothesis.

We then tested our first hypothesis of the effect of the inclusion of the PRPNF on the distribution of eye movements made to ROIs during encoding by comparing the eye movements of the new participants with those presented in the central location study. We tested this hypothesis by comparing the spatial and temporal distribution of eye movements across viewing time. In the central location study, the consistency of the spatial distribution of eye movements to ROIs across images was shown to be very high. We also report the same analyses for the present experiment. Doing so provides another index of the magnitude of the impact of the inclusion of the PRPNF on the distribution of eye movements across paintings.

The second hypothesis tested the effect of attending to the global or local Navon feature on the spatial distribution of the fixation in the varied location study. We also examined this due to the time course of sampling from each ROI during the encoding stage. Together, these analyses reveal the impact of prior viewing position on the accuracy and eye movements during the encoding stage.

We estimated the statistical power of the experiment on the basis of an effect size estimated from the most comparable existing study in the literature [39]. We used PANGEA (v0.2) [64]. The power of our current design was 0.855 for an effect size of d = 0.30. This value demonstrates that the study had sufficient power.

Data were processed in R version 3.5.0 [65]. Models were fitted using the lmer4 package [66] and MASS package [67]. The random effects were structured for items and participants including slopes for all fixed effects and correlation. The full random structure was trimmed down for those models that did not converge or had a correlation equal to zero or one. Specifically, for eye movement measures, the random structure for the LMM for the log-transformed normalised number of fixations was (1|Subject) + (1+ROIs|Stimuli) and for the log-transformed fixation duration as well as the log-transformed total fixation duration was (1|Subject) + (1|Stimuli)). The *t*-values equal to 1.96 or higher were interpreted as significant because for high degrees of freedom, the *t*-statistic in LMMs approximates the *z*-statistic [68].

### 3.1. Discrimination Accuracy

Response accuracy was treated as a binomial variable using the logistic General Linear Mixed Model (GLMM) in an intercept-only model. Fixed factors were structured for Study (2: varied location versus central location study) and Test Item (2: target versus foil). The reference levels were ‘central location’ and ‘target’.

**Figure 4:**
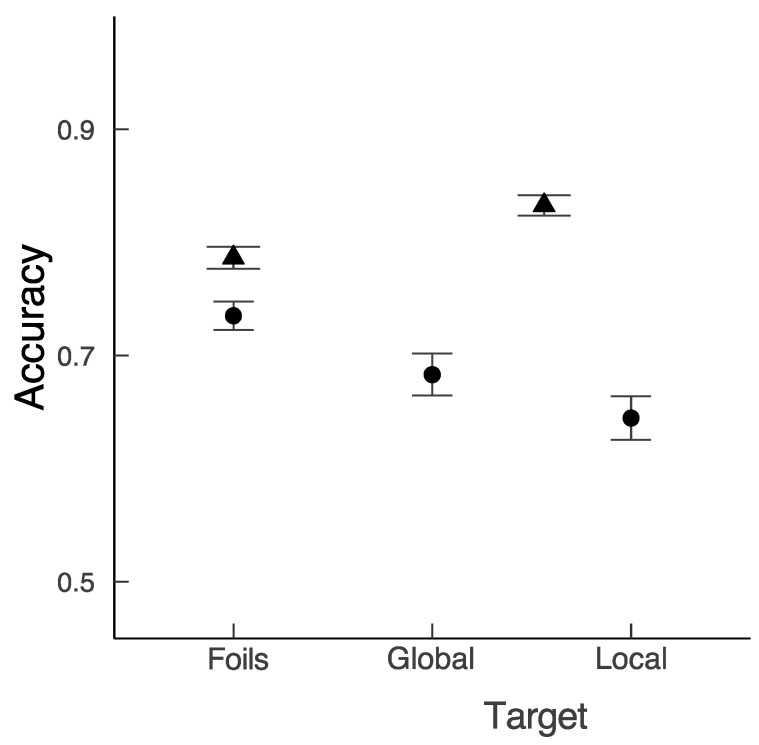
Mean accuracy as a function of Test Item for the varied location data and central location data. Note. Mean accuracy results for varied location study (circles) and comparable conditions from central location study (triangles). Error bars represent standard errors.

With respect to accuracy, the significant fixed effect of Study (*b* = −1.14, *SE* = 0.16, *z* = −7.04) and Test Item (*b =* −0.43, *SE* = 0.20, *z* = −2.12) was qualified by a significant interaction between Study and Test Item (*b =* −0.57, *SE* = 0.13, *z* = 4.24). While accuracy performance was higher in the central location study than in the varied location study, this difference was more pronounced for target paintings than foil paintings (see Figure 4).

To explore whether the spatial scale of the report of the PRPNF influenced accuracy, we computed an addition model with Test Item (2: global versus local versus foil) as a fixed factor for current data only. The reference level was ‘local’. The rejection of foils was better than the recognition of targets when participants reported the ‘local’ letter (*b =* 0.53, *SE* = 0.21, *z* = 2.51). By contrast, the difference in the recognition of targets presented in the local or global condition did not reach significance (*b =* 0.21, *SE* = 0.24, *z* = 0.38). Finally, for completeness, we used the Emmenas package version 1.4.5 [69] to create an additional comparison between foils and recognition of targets when participants reported the ‘global’ letter for the current model. The pairwise comparisons were corrected using Holm–Bonferroni correction and did not reach significance (*b =* 0.32, *SE* = 0.21, *z* = 1.50).

Overall, the discrimination accuracy data showed that varying the starting viewing position and spatial scale impaired performance. This was especially the case when the discrimination followed the local condition.

### 3.2. Eye Movements

The eye movement events were classified as fixations using the EyeLink data algorithm generated by Host Software’s online parses in real-time during recording, considering fixation as any period that is not a blink or saccade. The eye movement events were classified as saccades using an internal heuristic that applied a velocity- and acceleration-based detector. In addition, fixations that coincided with the display onset, response, or extreme outliers (<60 ms or >1200 ms fixation duration) were removed prior to data analysis. Only data from trials in which the PRPNF was correctly reported were analysed. Five participants’ data were excluded from the analysis due to an error rate of >25% on the PRPNF task. The remaining participants performed with high accuracy in both local and global conditions (*M* = 94%, *SE* = 1%; *M* = 95%, *SE* = 1%; respectively). The final data set consisted of 70,649 (out of 72,385) fixations in the encoding stage.

The normality of the eye movement measures was increased by log-transforming the data. Additionally, the number of fixations to ROIs was normalised by dividing the number of fixations to the ROI by the number of pixels within the area of the ROI to control for the differences in the spatial extent of regions across stimuli. Analyses were conducted on the normalised number of fixations, mean fixation duration, and total fixation duration.

#### 3.2.1. The Impact of Including the PRPNF on Spatial and Temporal Distribution of Eye Movements

To test the effect of the inclusion of the PRPNF on the distribution of eye movements during encoding, we first compared the eye movements of the varied location study with those of the central location study. Specifically, we examined fixation duration and saccade amplitude across the first 18 fixations (which approximated the first 4 s of viewing) to assess the processing time and distribution of eye movements across viewing time.

**Figure 5:**
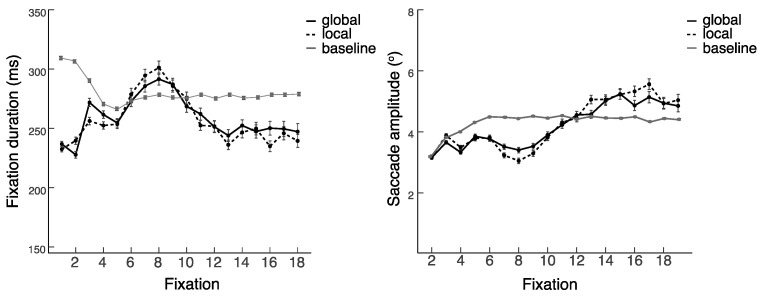
Mean fixation duration and saccade amplitude across the first eighteen fixations after painting onset for the varied location and central location study. *Note.* Error bars represent standard errors.

The inclusion of the PRPNF was associated with a period lasting around eight fixations in which participants engaged in relatively short fixations and saccades before a pattern of eye movements emerged, which was seen immediately upon the presentation of paintings in the absence of the PRPNF (see Figure 5). The pattern of speeding up fixations of lengthening saccade amplitude started from around the ninth fixation, when the paintings were preceded by the PRPNF, but it occurred from the first fixation, when the paintings were presented without the PRPNF.

We next explored the likelihood of making a fixation on the face, context, and theme ROIs and the proportion of fixations made on the face, context, and theme in the varied location study to those in the central location study. We conducted three intercept-only models (see Table 2). We did so to test the effect of disrupting the central fixation bias on the spatial distribution of eye movements made to ROIs during encoding. The fixed factor was structured for Study (2: varied location versus central location study). The reference levels were those for ‘central location’.

With respect to fixations on faces, participants were more likely to make a fixation on a face in the central location study than they were in the varied location study (*M =* 0.94, *SD =* 0.25; *M =* 0.13, *SD =* 0.33; respectively). Similarly, the proportion of fixations made on the faces (relative fixations made to the theme and context combined) was higher in the central location study than in the varied location study (*M =* 0.25, *SD =* 0.16; *M =* 0.08, *SD =* 0.06; respectively).

With respect to context and theme, there was no significant difference in the likelihood of making fixations to ROIs in the varied location study and the central location study. By contrast, participants made proportionally more fixations on the context and theme in the varied location and central location studies.

Together, these analyses suggest that the presentation of the PRPNF leads to a period of resetting in which participants seek to reset their eye movements with respect to memorising the painting. This period of resetting during viewing was associated with reduced fixations and the likelihood of making fixations on faces.

#### 3.2.2. Consistency of the Spatial Distribution of Eye Movements to ROIs across Images

**Figure 6:**
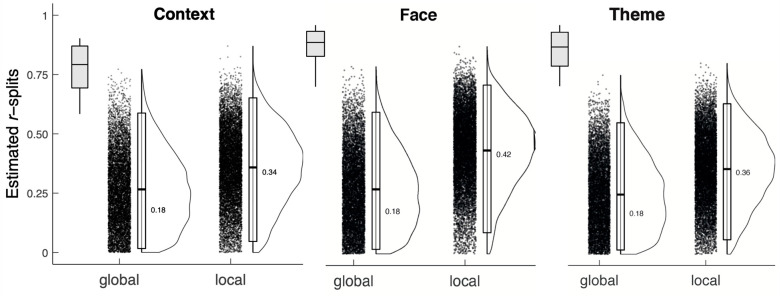
The bootstrapped split-half correlation for the proportion of viewing time of each ROI as a function of viewing condition. Note. The boxplot in each ROI indicates the default viewing condition reported by Trawiński et al. [39] for central fixation study.

The consistency of the proportion of total fixation duration made by participants to each ROI was examined using a permutation-based split-half approach with 10,000 random splits across all trials [70]. Trawiński et al. [39] reported an identical analysis for the same paintings and showed strikingly high consistency for the proportion of total fixation durations made to ROIs. The results from the varied location study are shown in Figure 6. The bootstrapped split-half correlations for the proportion of viewing time were relatively low.

The data suggest that the overall reliability of the proportion of fixations made on faces, themes, and context was greatly reduced by the inclusion of the PRPNF, and especially so when the participants attended to the global figure. The varied location data provide more evidence of the large impact of the inclusion of the PRPNF on the distribution of eye movements across paintings.

#### 3.2.3. The Impact of Attending to the Global or Local Navon Figure on Spatial and Temporal Distribution of Eye Movements

To test the impact of reporting the global or local PRPNF on eye movements in the varied location study only, the normalised mean number of fixations, mean fixation duration, and total fixation duration data were analysed with respect to two fixed factors: Condition (2: report of the global versus local Navon stimulus) and ROI (3: face versus context versus theme). The reference levels were global for Condition and face for ROI. The full LMM results are presented in Table 3.

**Figure 7:**
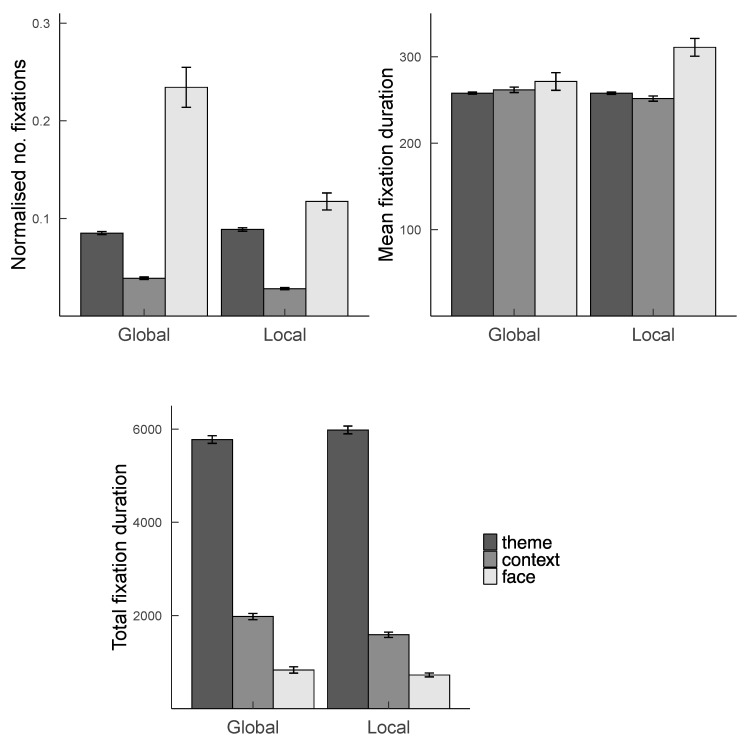
Mean normalised number of fixations, mean fixation duration, and total fixation duration as a function of ROI and condition in the encoding stage of varied location study. Note. Error bars represent standard errors.

Reporting the global PRPNF was associated with more, but shorter, fixations on faces than reporting the local PRPNF (see Figure 7). These effects combined such that there was no difference in the total duration of fixations on faces.

Overall, the results of the eye movement analyses show that the inclusion of the PRPNF delayed the adoption of the typical eye movement strategy with respect to fixation duration and saccade length and reduced the number and likelihood of fixations on faces, and the spatial scale attended influenced the number and duration of fixations on faces. The picture that emerges is that eye movements during the memorisation of paintings are markedly affected by the inclusion of the PRPNF.

**Figure 8:**
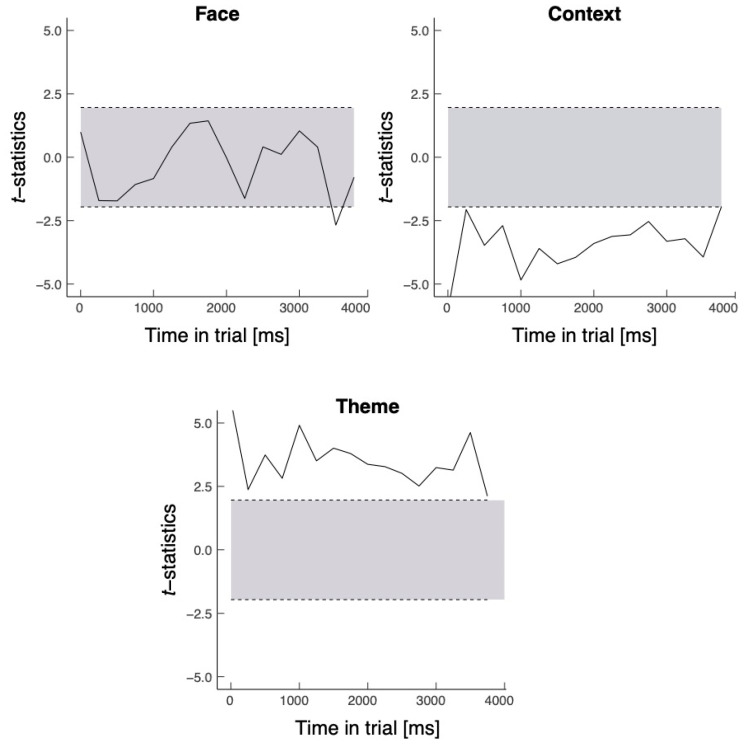
T-statistics, as a function of time in trial, for the time bin analysis. Note. Positive scores indicate a higher probability of looking at the specific ROIs in the local relative to the global condition. Area outside the shaded space indicates a significant difference.

Finally, we explored the time course of the probability of making fixations on each ROI over the first four seconds of viewing for all paintings presented during the encoding stage in the present study. The fixation data were aggregated across all participants into sixteen 250 ms time bins [71] to calculate the probability of fixating on the face, context, and theme [39]. To do so, time bin clusters were identified to estimate time windows of divergence between viewing conditions (see Figure 8).

Overall, the time course data show that the spatial scale of the PRPNF reported by the participants had an immediate and relatively long-standing impact on the eye movements made to the theme and context in the encoding stage. The need to report the global letter was associated with an increased likelihood of looking at the context. By contrast, the need to report the local letter was associated with an increase in looking at the theme.

In sum, the inclusion of the PRPNF was associated with a delay in the adoption of global eye movement behaviours previously found with the same images, reduced fixations on faces, increased fixations on the theme and context, and low consistency in eye movements across paintings. Furthermore, the spatial scale of the reported letter influenced the fixation strategy for memorising faces and the likelihood of fixating on the theme and context across the full duration of viewing.

## 4. Discussion

In the present study, we explored whether eye movements made to representational paintings when trying to memorise them were influenced by the prior presentation of a pseudo-randomly positioned Navon figure. The results we report suggest that eye movements made during the memorisation of paintings and the quality of encoding for recall are markedly affected by the inclusion of the PRPNF.

The inclusion of the PRPNF was associated with a delay in the eye movement strategy previously found with the same images. It took around eight fixations before a pattern of eye movements emerged akin to that previously reported with the same paintings. Specifically, after around eight fixations, participants tended towards shorter fixations and longer saccades. We interpret this finding as evidence of an attempt by participants to reset their eye movement after the offset of the PRPNF.

The need to reset eye movements had a marked influence on encoding. Relative to what we reported before in the central location study, the distribution of fixations across ROIs was shifted away from faces, such that the focus on the extent of faces was inconsistent across paintings. The inclusion of the PRPNF was disruptive to encoding such that the inclusion of the PRPNF reduced the accuracy of recognition relative to the data previously reported by [39].

The finding of disrupted encoding is probably related to other results reported in the context of scene inspection following the presentation of a mis-centred fixation cross (e.g., [44]). This literature demonstrates that eye movements following a mis-centred fixation cross show a short-term oscillatory pattern across scenes rather than being focused on the central area. Additionally, the effect of the substantial reduction in central fixation bias on viewing representational paintings accords with findings reported in previous scene perception studies [72,73]. Our results support the view that when the centre of the scene offers an optimal starting point for inspection, central fixation bias is optimal for task performance [32]. Specifically, by fixating on the centre of the image, viewers are able to encode visual information from the periphery of the scene through gist processing. By contrast, the amount of visual information being sampled is reduced when viewing begins at a location more towards the image boundary [74]. As a consequence of this reduced sampling, subsequent fixations are more likely to be located away from the centre [75].

It is also the case that a mis-centred fixation cross impacts the location of fixations to faces. For example, Or et al. [76] explored the influence of cultural background in fixations on faces. When guided by the centred fixation cross, an effect of culture on fixations on faces was found. However, the effect of culture on the location of fixations was removed when the mis-centred fixation cross was present at the beginning of the viewing. In sum, we think it would be worth exploring the effect of initial fixation position more systematically across different types of visual stimuli.

While the current data reflect the results of the studies conducted in laboratory conditions, they have implications for the understanding of spectatorship in a gallery setting. When the starting conditions for inspection cannot be controlled, the landing sites for the locations of initial fixations affect the beholder’s experience of viewing a painting. In particular, initial fixations that are made on the context risk lead to viewing dominated by an oscillatory pattern of eye movements across the paintings that does not support focus on the detailed information present in the theme or faces. The oscillatory eye movements are especially more likely to occur when spectators approach paintings while holding a global spatial scale.

When linking the low accuracy in the present task to the eye movement strategy adopted by the participants, it is important to acknowledge another possible explanation. The need to recall the global or local letter takes some capacity of the working memory, so that available for the encoding of paintings is reduced. If the inclusion of the PRPNF impacted the accuracy of the discrimination of old from new paintings by virtue of taking up significant resources in working memory, then one might expect to see a correlation between accuracy and working memory capacity. However, the correlation between working memory capacity and discrimination accuracy was not significant (*r* = −0.04 (95% CI: −0.44, 0.37) and *r* = 0.07 (95% CI: −0.35, 0.46) for 3-Back: Spatial and 3-Back: Verbal, respectively).

It is important to remember, when considering the present experiment and data, that participants were at liberty to view paintings for as long as they wished. The need to reset eye movements could have been factored in by participants such that they increased their overall fixation durations. The evidence shows that they did not do so and that encoding was taken as the outcome of the viewing strategy employed.

The spatial scale of the Navon figure that had to be reported had a profound and relatively long-lasting impact on eye movement. First, it impacted the pattern of fixations on faces. Second, and perhaps most importantly, it affected the likelihood of fixating on the theme and the context. Requiring participants to attend to the global spatial scale of the Navon figure meant that they were (pervasively) more likely to fixate on the context. By contrast, requiring participants to attend to the local spatial scale of the Navon figure meant that they were (pervasively) more likely to fixate on the theme. In interpreting these findings, it is important to note that the local and global scale scales of the Navon figure were in no way matched with the contents of specific paintings. The manipulation was much too coarse to make this claim. Rather, it seems likely that attending to the global spatial scale brings into focus features of the context that are more easily missed if attention is focused on a local detail in the theme. Once attended, these features continue to exert their influence on memory by expanding the area of inspection.

When interpreting the results of this study, we need to be cautious about its limitations. For example, the present study did not control for the effect of individual differences beyond visuo-cognitive abilities. There is evidence that personality [77] and culture (e.g., [39,56]) can influence eye movements. Moreover, we acknowledge that other individual differences may be important (e.g., sex, art expertise). The overall results reported in this study may be impacted by individual differences beyond those that we measured.

In conclusion, eye movements made when memorising paintings are subject to influence from the starting point of viewing and the spatial scale held by participants at the start of viewing. The findings of the present study are a reminder that it may be naïve to extrapolate from the eye movement patterns seen in response to paintings shown in the laboratory to those shown in a gallery setting. Moreover, varying the starting point for viewing and the viewers’ prior spatial scales were designed to reflect conditions that might influence spectatorship in a gallery. In other words, the experimental conditions were designed to reflect issues that might impact viewing in a gallery setting. The finding that the viewing conditions at play for beholders at the beginning of viewing very significantly influenced the memorisation of paintings suggests that gallery designers and curators should be aware of these factors. It may be that the conditions for viewing can be designed to optimise the beholder’s experience of viewing paintings.

## Figures and Tables

**Table 1:** The results of the battery of cognitive tests used to estimate the individual differences between the participants reported in the present study and those first reported in Trawiński et al. [39].

	Present Study		Trawiński et al.’s (2022) Study	
	*M*	*SD*	*M*	*SD*
3-Back: Spatial	57.87	11.38	52.53	18.96
3-Back: Verbal	60.03	11.12	61.18	21.37
ANT: EXEC	82.96	36.23	80.00	39.15
ANT: ALERT	25.78	38.62	33.18	32.78
ANT: ORIENT	24.66	37.24	36.92	43.46
A-S: Saccade Latency (1 s)	76.65	19.58	71.25	25.65
A-S: Saccade Latency (3 s)	83.15	23.35	81.62	36.58
Art Knowledge	8.32	4.25	8.51	5.42

Note. ANT = Attention Network Test; EXEC = executive; ORIENT = orienting; ALERT = alerting; A-S = Anti-Saccade task.

**Table 2:** Fixed-effect estimates from the linear mixed models for the likelihood of making fixations and the proportion of fixations on ROIs in the varied location and central location studies.

Predictors	Likelihood			Proportion of Fixations		
	*B*	*SE*	*z*	*b*	*SE*	*t*
Face						
Intercept	0.50	0.22	**2.27**	0.18	0.01	**13.31**
Study	3.45	0.15	**22.97**	0.06	0.01	**10.87**
Context						
Intercept	1.33	0.23	**5.70**	0.17	0.01	**12.91**
Study	−0.13	0.14	−0.92	−0.02	0.003	**−5.75**
Theme						
Intercept	12.68	2.53	**5.02**	0.74	0.01	**52.02**
Study	0.83	0.63	1.31	−0.09	0.01	**−18.14**

Note. Significant results are in bold.

**Table 3:** Fixed effect estimates from the linear mixed models for log-transformed normalised number of fixations, log-transformed mean fixation duration, and log-transformed total fixation duration on the type of ROI and condition in the encoding stage.

Predictors	Number of Fixations			Mean Fixation Duration			Total Fixation Duration		
	*B*	*SE*	*T*	*B*	*SE*	*T*	*b*	*SE*	*t*
Intercept	−2.34	0.11	**−20.67**	5.60	0.02	**234.06**	6.21	0.07	**84.14**
ROI (face vs. context)	−0.27	0.09	**−3.07**	−0.10	0.02	**−6.52**	0.75	0.05	**16.45**
ROI (face vs. theme)	0.24	0.09	**2.63**	−0.07	0.02	**−4.32**	2.35	0.05	**52.26**
Condition	−1.70	0.11	**−15.99**	−0.05	0.01	**−3.73**	0.004	0.04	0.10
ROI (face vs. context): Condition	−0.07	0.11	−0.67	0.07	0.02	**5.05**	0.12	0.05	**2.57**
ROI (face vs. theme): Condition	−0.26	0.09	**2.93**	0.06	0.02	**3.67**	−0.03	0.04	−0.63

Note. Significant results are in bold.

## Data Availability

The data presented in this study are available on request from the corresponding author.

## Supplementary Materials

The following supporting information can be downloaded at: https://www.mdpi.com/article/10.3390/vision7030055/s1, Table S1: List of paintings used at Encoding and Discrimination stage collapsed by authors and motifs.
