# Supplementary material for: The Effect of Prior Viewing Position and Spatial Scale on the Viewing of Paintings
Source: Vision (Basel). 2023 Aug 22;7(3):55. doi: 10.3390/vision7030055 (PMC10534493; doi:10.3390/vision7030055)
Supplement: Supplementary file 1 [file vision-07-00055-s001.zip › vision-2451452-supplementary.pdf]

Table S1: List of paintings used at Encoding and Discrimination stage collapsed by authors and motifs.

Author	Title	Year
	**Encoding stage**	
Baldung Grien, Hans	*The Three Graces*	c. 1540
Canova, Antonio	*The Three Graces Dancing*	c. 1799
Cranach, Lucas the Elder	*The Three Graces*	1535
Furini, Francesco	*The Three Graces*	c. 1633
Rubens, Peter Paul	*The Three Graces*	1639
Rafaello Sanzio	*The Three Graces*	1504
Rubens, Peter Paul	*Nature Adoring the Three Graces*	c. 1615
Botticelli, Sandro	*Primavera*	c. 1482
Tintoretto	*Mercury and the Graces*	c. 1576
Bronkhorst, Jan Gerritsz	*The Three Graces*	c. 1645
Moser, Koloman	*The Three Graces*	1905
Carle van Loo	*The Three Graces*	1763
Mathews, Arthur Frank	*Song of the Sea (Three Graces)*	c. 1909
Dali, Salvador	*Enchanted Beach With Three Fluid Graces*	1938
Delaunay, Robert	*La Ville de Paris*	1912
Scalbert, Jules	*The Three Graces dancing with Faun*	c. 1877
Janco, Marcel	*The Three Women in Malta*	1930
Fragonard, Jean_Honore	*The Three Graces*	1756
Etty, Wiliam	*Venus and Her Satellites*	1835
Picasso, Pablo	*Three woman*	1908
Botticelli, Sandro	*Judith Leaving the Tent of Holofernes*	c. 1495
Cairo, Francesco del	*Judith with Head of Holofernes*	c. 1645
Catena, Vincenzo	*Judith*	1520
Elsheimer, Adam	*Judith Beheading Holofernes*	1601
Gentileschi, Artemisia	*Judith and Her Maidservant*	c. 1614
Allori, Cristofano	*Judith with Head of Holofernes*	1613
Giorgione	*Judith*	c.1504

Riedel, August	*Judith*	1840
Rubens, Peter Paul	*Judith with Head of Holofernes*	c.1616
Tintoretto	*Judith and Holofernes*	c.1579
Tiziano	*Judith*	c. 1515
Klimt, Gustav	*Judith I*	1901
Valentin de Boulogne	*Judith*	c. 1626
Corot, Jean_Baptiste-Camille	*Judith*	c. 1872
Moser, Koloman	*Judith and Holofernes*	1916
Mellin, Charles	*Judith with Head of Holofernes*	1630
Piazzetta, Giovanni Battista	*Judith and Holofernes*	c. 1745
Cranach, Lucas the Elder	*Judith Victorious over Holofernes*	c. 1520
Carrachi, Agostino	*Juditt*	c.1590
Stuck, Franz	*Judith*	1928
Renoir, Pierre-Auguste	*Large Bathers*	c. 1884
Seurant, Georges	*Bathers at Asnieres*	c. 1883
Bazille, Jean-Frederic	*Bathers (summer Scene)*	1869
Vernet, Claude-Joseph	*Landscape with Bathers*	1783
Cezanne, Paul	*Bathers Beneath a Bridge*	c. 1895
Coubert, Gustave	*The Bathers*	1853
Gaugini, Paul	*The Bathers*	1897
Fragonard, Jean-Honore	*The Bathers*	c. 1772
Carracci	*Landscape with Bathers*	1616
Cezanne, Paul	*The Large Bathers*	c. 1900
Kirchner, Ernst Ludwig	*Bathers at Mortizburg*	c. 1909
Cezanne, Paul	*Bathers*	c. 1872
Cezanne, Paul	*Bathers*	c. 1890
Andre Derain	*Bathers*	1907
Picasso, Pablo	*Bathers with Toy Boat*	1937
Picasso, Pablo	*Bathers*	1918
Picasso, Pablo	*Les Demoiselles d'Avignon*	1907
Walker, Frederick	*The Bathers*	c. 1866
Matisse, Henri	*Joy of Life*	c. 1905
Matisse, Henri	*Bathers with turtle*	1908
Leighton, Frederic	*Odalisque*	1862

Boucher, Francois	*Brown Odalisque*	1745
Delacroix, Eugene	*Odalisque*	1857
Ingres, Jean-Auguste-Dominique	*The Grand Odalisque*	1814
Renoir, Pierre-Auguste	*Odalisque*	1870
Matisse, Henri	*Odalisque, Harmony in Red*	c. 1926
Tanoux, Adrien Henri	*Odalisque*	1913
Schiovoni, Natale	*Odalisque*	1845
Matisse, Henri	*Odalisque*	1926
Picasso, Pablo	*The Great Odalisque (after Ingres)*	1907
Picou, Henri Pierre	*Odalisque*	1858
Picasso, Pablo	*Woman of Algier (Version N)*	1955
Picasso, Pablo	*Jacqueline in Turkish Dress*	1955
Corot, Jean_Baptiste-Camille	*The Roman Odalisque*	1843
Fabbi, Fabio	*Girls of the Harem*	c. 1906
Delacroix, Eugene	*The Women of Algiers in Their*	1834
Jonghe, Gustave Leonard	*A reclining Odalisque*	c. 1870
Fortuny, Maria	*The Odalisque*	1861
Lefebvre, Jules Joseph	*Odalisque*	1874
Bukovac, Vlaho	*Odalisque*	1882
Botticelli, Sandro	*The Birth of Venus*	1486
Cabanel	*The Birth of Venus*	1683
Fauconnet, Guy Pierre	*Venus*	1919
Titian	*The Venus of Urbino*	1538
Picasso, Pablo	*Nude woman with Necklece*	1968
Cranach, Lucas the Elder	*Cupid Complaining to Venus*	1525
Sustris, Lambert	*Venus and Love*	1550
Matisse, Henri	*Venus*	1952
Rosetti, D. G.	*Venus*	c. 1863-
Velazques, Diego	*Venus at her Mirror*	1601
Gossart, Jan	*Venus*	c. 1521
Rubens, Peter Paul	*Venus at a Mirror*	c. 1615
Modigliani, Amadeo	*Venus-Maja*	1917
Rembrandt van Rijni	*Hendrickje Stoffels as Venus*	1662

Albani, Francesco	*Venus Attended by Nymphs and Cupids*	1633
Bollandt, Heinrich	*Venus and Amor*	c. 1520
Lambert, Sustris	*Venus and Love*	1550
Boucher, Francois	*The Triumph of Venus*	1740
Ingres, Jean-Auguste-Dominique	*Venus Anadyamene*	c. 1825
Dali, Salvador	*Venus Binding Cupids*	1925
	**Discrimination stage**	
Aachen, Hans von	*The three Graces*	1604
Bisson, Eduard	*The Three Graces*	1899
Bouvier, Jules Augustus	*The Three Graces*	1875
Cranach, Lucas the Elder	*The Three Graces*	1531
Delaunay, Robert	*The Three Graces*	1912
Frost, William	*The Three Graces*	c. 1854
Picasso, Pablo	*The Three Graces*	1908
Picasso, Pablo	*The Three dancers*	1925
Vernon, Emile	*The Three Graces*	1917
Rubens, Peter Paul	*The Three Graces*	1620
Botticelli, Sandro	*Primavera*	c. 1482
Bronchorst, Jan Gerritsz	*The Three Graces*	c. 1645
Dali, Salvador	*Enchanted Beach With Three Fluid Graces*	1938
Etty, Wiliam	*Venus and Her Satellites*	1835
Furini, Francesco	*The Three Graces*	c. 1633
Janco, Marcel	*The Three Women in Malta*	1930
Mathews, Arthur Frank	*Song of the Sea (Three Graces)*	c. 1909

Picasso, Pablo	*Three woman*	1908
Rubens, Peter Paul	*The Three Graces*	c. 1615
Tintoretto	*Mercury and the Graces*	c. 1576
Botticelli, Sandro	*The return Judith to Bethulia*	1427
Carravagio	*Judith Beheadinng Holofernes*	c.1598
Cranach, Lucas the Elder	*Judith Victorious*	c.1530
Gentileschi, Artemisia	*Judith and Holofernes*	1620
Goya, Francisco	*Judith and Holofernes*	1819
Klimt, Gustav	*Judith II*	1909
Lama, Gulia	*Judith and Holofernes*	1730
Vasari, Giorgio	*Judith and Holofernes*	c. 1554
Bray, Salomon de	*Judith Delivering the Head of Holofernes*	1636
Vermeyen, Jan Cornelisz	*Judith with Head of Holofernes*	c. 1525
Botticelli, Sandro	*Judith Leaving the Tent of Holofernes*	c. 1495
Cairo, Francesco del	*Judith with Head of Holofernes*	c. 1645
Corot, Jean_Baptiste-Camille	*Judith*	c. 1872
Giorgione	*Judith*	c.1504
Moser, Koloman	*Judith and Holofernes*	1916
Mellin, Charles	*Judith with Head of Holofernes*	1630
Riedel, August	*Judith*	1840
Piazzetta, Giovanni Battista	*Judith and Holofernes*	c. 1745
Stuck, Franz	*Judith*	1928

Valentin de Boulogne	*Judith*	c. 1626
Picasso, Pablo	*Bathers in Forest*	1908
Wouwerman, Philips	*Landscape with Bathers*	c.1660
Cezanne, Paul	*Bathers*	1892
Gaugini, Paul	*Bathers at Tahiti*	1897
Kirchner, Ernst Ludwig	*Three Bathers*	1913
Peter, Jean Baptiste Joseph	*The Bathers*	c. 1721
Preisler, Jan	*Bathers*	1912
Renoir, Pierre-Auguste	*The Bathers*	1918
Seurat, Georges	*Study for Bathers at Asnieres*	1883
Cezanne, Paul	*Bathers*	c. 1900
Bazille, Jean-Frederic	*Bathers (Summer Scene)*	1869
Carracci	*Landscape with Bathers*	1616
Cezanne, Paul	*The Large Bathers*	c. 1900
Fragonard, Jean-Honore	*The Bathers*	c. 1772
Walker, Frederick	*The Bathers*	c. 1866
Gaugini, Paul	*The Bathers*	1897
Matisse, Henri	*Joy of Life*	c. 1905
Picasso, Pablo	*Bathers with Toy Boat*	1937
Picasso, Pablo	*Bathers*	1918
Courbet, Gustave	*The Bathers*	1853
Boucher, Francois	*Blond Odalisque*	1752
Ingres, Jean-Auguste-Dominique	*Odalisque with slave*	1842

Matisse, Henri	*Odalisque with a Green Plant and Screen*	1923
Matisse, Henri	*Reclining Odalisque*	1926
Picasso, Pablo	*Femmes d'Alger*	1955
Renoir, Pierre-Auguste	*Parisian Women in Agerian Costume*	1872
Tanoux, Adrien Henri	*Odalisque*	1904
Weisz, Adolphe	*Odalisque*	1884
Gervex, Henri	*Odalisque*	1882
Renoir, Auguste	*Reclining Odalisque*	c. 1917
Bukovac, Vlaho	*Odalisque*	1882
Corot, Jean_Baptiste-Camille	*The Roman Odalisque*	1843
Delacroix, Eugene	*The Women of Algiers in Their*	1834
Ingres, Jean-Auguste-Dominique	*The Grand Odalisque*	1814
Lefebvre, Jules Joseph	*Odalisque*	1874
Leighton, Frederic	*Odalisque*	1862
Picasso, Pablo	*Jacqueline in Turkish Dress*	1955
Picasso, Pablo	*The Great Odalisque (after Ingres)*	1907
Picou, Henri Pierre	*Odalisque*	1858
Schiovoni, Natale	*Odalisque*	1845
Amaury, Duval	*La Naissance de Venus*	1862
Bouguereau, A.	*The Birth of Venus*	1879
Picasso, Pablo	*Venus et L'Amour*	1957
Giorgione	*Sleeping Venus*	c. 1510
Titian	*Venus and Music*	1547

Rubens, Peter Paul	*Venus Frigda*	1614
Girodet de Roucy-Trison, Louis	*Mademoiselle Lange as Venus*	1798
Tintoretto	*Venus, Mars and Vulcan*	c. 1551
Carracci	*Sleeping Venus*	c. 1602
Poussin, Nicholas	*Venus and Satyr*	1626
Boucher, Francois	*The Triumph of Venus*	1740
Dali, Salvador	*Venus Binding Cupids*	1925
Fauconnet, Guy Pierre	*Venus*	1919
Gossart, Jan	*Venus*	c. 1521
Matisse, Henri	*Venus*	1952
Modigliani, Amadeo	*Venus-Maja*	1917
Picasso, Pablo	*Nude woman with Necklace*	1968
Rembrandt van Rijni	*Hendrickje Stoffels as Venus*	1662
Rubens, Peter Paul	*Venus at a Mirror*	c. 1615
Sustris, Lambert	*Venus and Love*	1550

Note. In fourth column is shown motif categories (1 = *Three Graces*, 2 = *Judith,* 3 = Bathers, 4 = *Odalisque*, 5 = *Venus*).
